# Retinal detachment in patients with Sticklers syndrome: A comprehensive analysis for craniofacial surgeons

**DOI:** 10.1016/j.jpra.2026.01.017

**Published:** 2026-01-27

**Authors:** Annelie J. Bleeker, Nathaniel A.T. Sullivan, Anna H. Brouwer, Elizabeth J. de Koster, Michelle B. van Egmond-Ebbeling, Elsbeth S.M. Voskuil-Kerkhof, Peter A.W. Schellekens, Marie-José H. van den Boogaard, Corstiaan C. Breugem

**Affiliations:** a Department of Ophthalmology, University Medical Center Utrecht, Utrecht, the Netherlands; b Department of Ophthalmology, Deventer Hospital, Deventer, the Netherlands; c Department of Pediatric Plastic and Reconstructive Surgery, University Medical Center Amsterdam, Amsterdam, the Netherlands; d Department of Clinical Genetics, University Medical Center Utrecht, Utrecht, the Netherlands; e Department of Radiology and Nuclear Medicine, Radboud University Medical Centre, Nijmegen, the Netherlands; f Department of Ophthalmology, Eyescan, Ede, the Netherlands

**Keywords:** COL2A1, Hereditary connective tissue disorders, Robin sequence, Retinal detachment, Risk factors, Stickler syndrome

## Abstract

**Background:**

Stickler syndrome (SS) is the leading cause of hereditary retinal detachment (RD) in children and is characterized by ophthalmic, auditory, orofacial, and articular abnormalities. Vision loss often results from retinal events (RE), including retinal tears and detachments.

**Purpose:**

To identify clinical and genetic risk factors for retinal events in patients with SS, to guide screening and preventive care.

**Methods:**

This retrospective cohort study included 78 patients with clinically or genetically confirmed SS seen at the University Medical Center Utrecht between 2000 and 2019. Predictors included family history of SS, presence of a COL2A1 pathogenic variant, and degree of myopia. The primary outcome was occurrence of a retinal event, defined as a retinal tear or detachment confirmed by an ophthalmologist. Covariates were age, sex, Pierre Robin sequence, refractive error, retinopathy, hearing problems, joint problems, and cleft palate. Descriptive statistics, univariate analyses, and multivariate Kaplan–Meier survival models with multiple imputation and Firth’s correction were used.

**Results:**

Nineteen of 78 patients (24%) developed at least one retinal event at a median age of 14 years. Multivariate analysis revealed that a positive family history of SS (HR 5.53, 95% CI 1.42–21.55), COL2A1 pathogenic variant (HR 3.59, 95% CI 1.00–12.82), and greater myopic refractive error (HR 0.86, 95% CI 0.74–0.99) were significantly associated with increased RE risk.

**Conclusion:**

A positive family history, COL2A1 mutation, and higher myopia independently predict retinal events in Stickler syndrome. Regular ophthalmologic screening is recommended for younger and high-risk patients, while education on early symptoms of retinal detachment is essential for older children and adults.

## Introduction

Pediatric rhegmatogenous retinal detachment (RD) is a rare condition with an incidence ranging from 0.38 to 0.69 per 100,000 people per lifetime, with Stickler syndrome (SS) being the most common cause.^1^ Early recognition of SS and prompt detection of RD are crucial for preserving vision in affected individuals. SS, a heterogeneous group of hereditary connective tissue disorders, has an estimated prevalence of 11.1 to 13.3 per 100,000 persons.^2^ It is characterized by ophthalmic, auditory, orofacial, and articular manifestations, with ophthalmological features including vitreous changes, retinal abnormalities, and high myopia. A significant complication of SS is the development of retinal tears, which can progress to RD.

The inheritance pattern of SS can be either autosomal dominant or autosomal recessive, exhibiting complete penetrance but variable expressivity. The genetic landscape of SS is complex and evolving. While earlier literature described six genes associated with SS: COL2A1, COL11A1, COL11A2, COL9A1, COL9A2, and COL9A3,^3^ recent studies have expanded this list. Nixon et al. (2018) and subsequent research have identified additional genes and genetic mechanisms involved in SS.^4^ The reported risk for RD in SS patients varies considerably, ranging from 38% to 70%.^5^ This wide range may be attributed to the different subtypes of SS and their associated genetic mutations. Additionally, some rare families may harbor mutations in currently unknown genes, while biallelic variants in LOXL3 and LRP2 have been reported as candidate genes in certain cases.^6^^,^^7^ It is worth noting that pathogenic variants in the VCAN gene cause Wagner syndrome, which shares overlapping ocular symptoms with SS.^8^

The clinical or genetic diagnosis of SS is often initiated by the identification of affected family members or during the evaluation of patients with other congenital anomalies associated with SS, such as Robin sequence (RS).^9^ RS is a disorder characterized by a sequence of micrognathia, glossoptosis, and upper airway obstruction. In addition to these primary characteristics, approximately 90% of individuals with RS also present with a cleft palate. RS can manifest in two forms: isolated or as part of a broader genetic syndrome, such as SS. This distinction is crucial for proper diagnosis, management, and genetic counseling, as the underlying causes and associated features may differ between isolated and syndromic cases.^10^^,^^11^

The varying range in reported risk for RD is SS patients may be attributed to the different subtypes of SS and their associated genetic mutations. Patients with COL2A1 pathogenic variants, pre-existent vitreous changes, and lattice degeneration are thought to have a higher risk for RD.^3^^,^^12^, ^13^, ^14^, ^15^ However, a comprehensive multivariate analysis exploring potential colinearities between these risk factors is currently lacking in the literature.

Treatment for most SS patients with RD typically involves scleral buckling or a combination of scleral buckling with vitrectomy.^16^ Prophylactic therapy of the fellow eye using scleral buckling, cryotherapy, or laser has been reported as effective in retrospective studies.^3^ Nevertheless, higher-level evidence from prospective and/or randomized trials is not yet available to confirm these findings. Furthermore, more personalized treatment strategies based on genetic diagnosis could potentially improve outcomes.

To mitigate the risk of vision loss in SS patients, the USA SS protocol recommends annual ophthalmological screenings and advises limiting participation in activities with a high risk of traumatic RD, such as contact sports.^5^ However, the evidence base supporting these recommendations is limited. There is a notable absence of multivariate analyses that could provide deeper insights into the clinical or genetic risk factors for RD at a young age in SS patients. This gap in knowledge is particularly significant because young patients may not reliably report symptoms of RD.

In light of these limitations, our current study aims to address these knowledge gaps by describing a consecutive cohort of SS patients. We report the incidence of retinal events (RE; either detachment or tears) and employ a multivariate model to identify risk factors for RE in children with SS. This approach seeks to provide a more comprehensive understanding of the factors influencing retinal complications in SS, potentially informing more targeted screening and preventive strategies, especially for younger patients who may be at higher risk.

## Methods

### Study design and patient population

A retrospective cohort study was conducted, encompassing all consecutive SS patients who sought consultation at the ophthalmological department of our tertiary referral center, the University Medical Center Utrecht, Netherlands, between January 2000 and September 2019. For this study, SS patients were defined as those with either a clinical or genetic diagnosis of the condition. Clinical diagnosis was established based on the diagnostic criteria defined by Rose; which include: ocular findings (myopia, vitreoretinal degeneration, retinal detachment), hearing loss, joint hypermobility or arthritis, cleft palate or micrognathia; where a clinical diagnosis of SS is made if a patient exhibited at least two of the five major diagnostic criteria.^9^ In some instances, the clinical diagnosis of SS was solely based on ophthalmological signs observed by an ophthalmologist, without further general or genetic counseling. Genetic testing was performed using a specialized SS gene panel, which included COL2A1, COL9A1, COL9A2, COL9A3, COL11A1, COL11A2, LRP2, and VCAN. Notably, patients with COL11A2 pathogenic variants were excluded from the current study due to the absence of ocular symptoms in the associated diagnosis. Ethical approval for this study was obtained from the medical ethical research committee of our institution, and informed consent was not required for this retrospective analysis.

### Patient management

At our center, pediatric SS patients undergo ophthalmological screening every 6 months, beginning either at birth or at the time of diagnosis. This screening regimen typically continues through adolescence, although a standardized protocol defining its precise duration is currently lacking. When a RD occurs, the option of prophylactic treatment for the contralateral eye is discussed with the patient and their parents, with considerations tailored to the patient’s age. In contrast to the pediatric approach, adult SS patients generally do not receive periodic screenings. Instead, these patients are thoroughly educated about the symptoms of RD and instructed to seek prompt ophthalmological evaluation should such symptoms manifest.

### Data analysis

All data relevant to our research question was extracted from patients’ medical files, including age, sex, genotype, family history, association with RS, cleft palate, history of deafness, joint problems, ophthalmological signs of vitreous anomalies, retinopathy, refractive error, and ophthalmological outcomes of RD and retinal tears. Vitreous anomalies were defined as described by Snead. Myopia was classified as “high myopia” if a spherical refractive error ≥−5.0 diopters was present, in accordance with the World Health Organization definition.

All RE per patient were recorded, with a RE defined as a retinal tear or RD in one eye, diagnosed by an ophthalmologist. Age at the time of RE was noted, and patients with missing data regarding their age at the time of their event were excluded. End of follow-up was defined as the last ophthalmological visit at the date of inclusion.

Due to small subgroup sizes, patients were categorized into two groups for comparison (Table 5). The first group included clinically diagnosed SS patients with genetically confirmed PV in COL2A1, and clinically diagnosed SS patients not yet genetically confirmed but with affected family member(s) with genetically confirmed COL2A1 pathologic variants. The second group encompassed all other identified SS patients, aggregating three subgroups: clinically diagnosed SS patients genetically not confirmed after up-to-date genetic testing, SS patients with genetically confirmed PVs in COL9A1, COL9A2, COL9A3, COL11A1, and clinically diagnosed SS patients without genetic testing.

### Statistical analysis

For baseline characteristics, descriptive statistics were applied using the mean ± standard deviation or the median with interquartile range for continuous data, depending on whether the data followed a normal or non-normal distribution. Categorical data were summarized as absolute and relative frequencies expressed as percentages. Univariate between-group comparisons of baseline characteristics were conducted using independent sample T-tests for continuous variables and Chi-squared tests for categorical variables. Age was excluded from the univariate analysis due to the cross-sectional design of the study.

Subsequently, multivariate Kaplan-Meier analysis was employed to compare SS patients with and without RE, utilizing patients’ age at the time of the event or at the end of follow-up. To address potential data loss in this small study population, multiple imputation techniques were implemented. Firth’s correction was applied to mitigate small sample bias. Variables included in the multivariate analysis were selected based on their statistical significance in univariate analysis and/or their clinical relevance as supported by existing literature. A *p*-value of ≤ 0.05 was considered statistically significant in all analyses. Univariate analyses were performed using IBM SPSS Statistics version 25.0 (IBM Corp, Armonk, NY, USA), while multivariate analyses were conducted using SAS Enterprise Guide version 9.4 (SAS Institute Inc., Cary, NC, USA).

## Results

A total of 83 SS patients were initially identified for this study. Following the exclusion of three patients with COL11A2 PVs and two patients with missing age data at the time of their event, 78 patients were ultimately included in the analysis. Table 1 presents the baseline characteristics of the study cohort.

**Table 1:** Patient characteristics.

Characteristic	*n* (%)	median (IQR)
Age at end of follow-up in years		17 (9–30)
Female	51 (65%)	
COL2A1 pathologic variant^a^	35 (45%)	
Family history of Sticklers syndrome	35 (45%)	
Spherical refractive error		S-4.25 (S-9.50–S-0.25)
Cylindrical refractive error		C-1.50 (C-2.50–C-0.50)
Vitreous anomaly^b^	33 (42%)	
Retinopathy^c^	33 (42%)	
Pierre Robin sequence	20 (26%)	
Cleft palate	33 (42%)	
Hearing problems	18 (23%)	
Joint problems	19 (24%)	
Outcome		
Retinal detachment	18 (23%)	
Retinal tear without detachment	1 (1%)	
Age in years at first retinal event^d^		14 (10–24)

a Consists of both clinically SS patients with genetically confirmed COL2A1 pathogenic variant and (not) yet genetically confirmed SS patients, but with COL2A1 pathogenetic variant segregating in the family.

b According to Snead et al. with membranous congenital vitreous, beaded congenital vitreous, or empty vitreous.

c i.e., mention of lattice degeneration or retinal schisis.

d A retinal event was defined as a retinal tear or retinal detachment in one eye, diagnosed by an ophthalmologist.

Genetic testing results revealed COL2A1 PVs in 35 (45%) patients and COL11A1 PVs in 2 (3%) patients. No PV was identified by gene panel testing in 19 (24%) patients. The remaining 22 (28%) patients did not undergo genetic screening and were diagnosed with SS based on clinical characteristics. Table 1 of the supplemental digital content provides more detailed information on all pathologic variants.

A univariate comparison of baseline characteristics between the 37 (47%) genetically confirmed SS cases and the 41 (53%) clinically diagnosed SS patients yielded significant findings. Genetically confirmed cases demonstrated a higher frequency of RS (39% vs. 17%, *p* = 0.04) and a significantly younger age at RE or end of follow-up (median 11 vs. 14 years, *p* = 0.02).

Of the total cohort, 19 (24%) patients experienced at least one RE requiring treatment. Among these, 18 patients developed at least one RD, while one patient presented with a retinal tear. The median age at first RE was 14 years (IQR 10–24 years), with four patients being younger than 10 years of age. The group of patients with RD exhibited considerable variability in follow-up time prior to their RE. Six (32%) patients underwent screening before their first RE, while 8 (42%) patients only initiated screening after their RE. The remaining 5 (26%) patients had an ophthalmological history at another hospital and were subsequently screened at our tertiary care center following treatment for a RE.2 (33%) patients that were screened in advance of their RE had RS and had vitreous anomalies reported, but without retinopathy prior to their RE. In the remaining 4 (67%) patients without RS, retinopathy was previously reported, including vitreous anomalies in 3 of the 4 (75%) patients. Retinopathy in these cases included radial or perivascular lattice degeneration and vitreous abnormalities typical of SS.

Univariate comparison between 35 (45%) SS patients with a COL2A1 PV to the other 43 (55%) SS patients indicated that RS occurred more frequently in patients with a COL2A1 PV (mean 0.40 ± 0.50) compared the other SS patients (mean 0.16 ± 0.37, *p* < 0.001). Besides RS, no other statistically differences were found between these two groups.

Univariate comparison between patients with and without a RE showed that patients with a RE more often had signs of retinopathy (*p* = 0.000) and high myopia (*p* = 0.01) (Table 2).

**Table 2:** Retinal events.

Characteristic	Retinal event^a^		*p*-value
	Yes	No	
	(*n* = 20)	(*n* = 58)	
Female (*n* = 51 of 78)	12 (63%)	39 (66%)	0.82^b^
COL2A1 pathogenic variant (*n* = 35 of 78)	10 (53%)	25 (42%)	0.43^b^
Family history of Sticklers syndrome (*n* = 35 of 78)	10 (50%)	25 (43%)	0.60^b^
Myopia (*n* = 56 of 78)	17 (90%)	39 (66%)	0.05^b^
Highest spherical refractive error (median, *n* = 73 of 78)	−8.75D	−2.75D	0.01^c^
Vitreous anomaly (*n* = 33 of 77)	10 (53%)	23 (40%)	0.32^b^
Retinopathy (*n* = 33 of 78)	17 (90%)	16 (27%)	0.000^b^
Pierre Robin sequence (*n* = 20 of 73)	4 (24%)	16 (29%)	0.68^b^
Cleft palate (*n* = 33 of 72)	4 (25%)	29 (52%)	0.06^b^
Hearing problems (*n* = 18 of 73)	2 (12%)	16 (29%)	0.16^b^
Joint problems (*n* = 19 of 73)	4 (24%)	15 (27%)	0.79^b^

a A retinal event was defined as a retinal tear or retinal detachment in one eye, diagnosed by an ophthalmologist.

b Chi-square test.

c Independent-samples median test.

Some patients had missing values. Five (6%) patients had unknown refractive error. Signs of hearing problems or joint problems were unknown in 5 (6%) patients. Medical files of 6 (8%) patients lacked information about the presence of a cleft palate. After imputation of missing values, multivariate analysis was performed including the following parameters: age at the time of a RE or at the end of follow up, sex, gene PV COL2A1, highest spherical refractive error, highest cylinder, family history of Stickler, occurrence of RS, retinopathy, hearing problems, joint problems, and cleft palate (Table 3).

**Table 3:** Multivariate survival analysis.

Characteristic	Theta0	*p*-value	Hazard ratio [95% CI]
Female	−0.74	0.46	0.60 [0.16–2.32]
Family history of Stickler	2.46	0.01	5.53 [1.42–21.55]
Pierre Robin sequence	−0.56	0.58	0.52 [0.05–5.24]
Highest spherical refractive error	−2.09	0.04	0.86 [0.74–0.99]
Highest cylindrical refractive error	1.04	0.30	1.30 [0.79–2.15]
Retinopathy	1.60	0.11	3.54 [0.75–16.66]
Hearing problems	−1.27	0.21	0.33 [0.06–1.84]
Joint problems	−0.34	0.74	0.81 [0.23–2.80]
Cleft palate	0.35	0.73	1.53 [0.14–16.26]
Genetic COL2A1 mutation	1.96	0.05	3.59 [1.00–12.82]

In our multivariate survival analysis, SS patients with a RE significantly more often had family members affected with SS, both clinically and genetically confirmed (hazard ratio (HR) 5.53 [95% CI 1.42–21.55]). Patients with a COL2A1 PV also had a higher HR compared to patients who did not (3.59 [95% CI 1.00- 12.82]). A higher spherical refractive error was also significantly associated with the occurrence of a RE in SS patients as shown by the negative HR of 0.86 [95% CI 0.74–0.99] (Supplemental Digital Content 2 & 3).

## Discussion

SS is a relatively rare disease with a high occurrence of ocular complications. In our cohort, we observed a RE in 24% of SS patients. The median age at time of RE was 14 years, with the youngest patient being only 5 years old. We identified the following risk factors for RD in SS patients: a positive family history of SS, carrying a COL2A1 PV, and a myopic refractive error >5.

During genetic counseling, 35 (45%) of our patients were diagnosed with a COL2A1 PV, which is comparable to the 49% reported by Boysen et al., which is a large review of 1417 SS patients.^3^ We anticipate a higher incidence of COL2A1 PVs if genetic testing were performed on the 22 (28%) patients who were only clinically diagnosed with SS. The age at the time of RE in our study (median 14 years) was also consistent with Boysen et al., who reported a mean age between 10 and 20 years. The incidence of RD in SS patients with COL2A1 PV in our study (53%) was slightly lower than previously reported incidences (60–73%). In the systematic review by Boysen et al. there was no significant difference in risk of RD within different gene PVs.^3^

The incidence of RE in our study (24%) was lower than the 45% previously described by Boysen et al. in A possible explanation could be our cross-sectional study design and the young median patient age of 17 years (IQ range 9–30). Therefore, we assume that the lifetime risk of RE will be higher than the 24% we found.

Positive family history as a risk factor for RD is also mentioned by Edwards et al., who described two COL2A1 pedigrees with a predominantly ocular phenotype.^17^ This can be explained by the specific genetic PVs present in one family.

Our study presents both strengths and limitations. To our knowledge, it is one of the largest cohort studies to investigate the prevalence of RE’s in SS patients. While there is growing understanding of the genotypes and specific PVs associated with SS, it remains primarily a clinical diagnosis. Therefore, we included both genetically confirmed SS patients and those diagnosed clinically.

We employed Kaplan-Meier analysis to adjust for varying follow-up periods within the cohort. To address potential collinearity among variables, we conducted a multivariable analysis. Multiple imputation was utilized to mitigate data loss in our small study population, and Firth’s correction was applied to minimize small sample bias. Despite these efforts, the total number of patients was relatively small for conducting a robust statistical analysis.

Multivariate survival analysis revealed no significant association with retinopathy, as observed in univariate analysis, possibly due to reporting bias. Approximately one-third of the patients underwent screening at our hospital prior to the RD, with only a few having previously documented retinopathy. Thus, retinopathy is often reported at the time of RD diagnosis, although it likely existed beforehand.

Our study has several limitations that should be considered when interpreting the results. The retrospective design and relatively small sample size limit the statistical power of our analyses. The inclusion of patients without genetic confirmation may introduce heterogeneity into the study population. Future prospective studies with larger cohorts and comprehensive genetic testing are needed to validate our findings and provide more definitive guidance on screening protocols for SS patients.

In our study cohort, a significant proportion of patients clinically diagnosed with SS did not undergo genetic screening, accounting for 28% (22 patients) of the total group. This subset of patients potentially harbors a higher incidence of COL2A1 mutations, which could be revealed through genetic testing. Genetic confirmation of SS was successfully achieved in 47% of the cohort (37 patients), who were characterized by younger age and a higher frequency of RS. This observation suggests a correlation between the severity of clinical manifestations and earlier age of presentation, as well as an increased likelihood of receiving genetic counseling. The higher rate of genetic confirmation in this group may be attributed to the preferential request for genetic counseling in patients presenting with cleft palate compared to those with ophthalmological symptoms such as RD or clinical suspicion of SS. These findings emphasize the critical role of comprehensive genetic screening in SS diagnosis and highlight potential biases in patient selection for genetic testing based on clinical presentation and symptom severity. High axial length of the eye is recognized as a significant contributor to RD. However, in routine clinical practice, axial length measurements are infrequently performed outside the context of cataract surgery. To address this limitation in our study, we incorporated spherical refractive error as a proxy measure. This decision was informed by the well-established correlation between refractive error and axial length, with spherical refractive error serving as a reasonable indicator of axial length.^18^

In Stickler, the myopia is largely congenital rather than environmentally driven. Reviews on environmental factors in myopia specifically point out that for genetic myopias the usual advice about near work and outdoor time is unlikely to have much effect because eye size and vitreous phenotype are determined by the underlying collagenopathy. By contrast, the strong evidence for outdoor time/less near work comes from common/axial school-age myopia, not congenital high myopia. Extrapolating it to SS would be speculative at best. Researching this retrospectively cannot be done as exposure to distance viewing is not commonly noted in ophthalmology notes.

Internationally, several strategies for RD prevention and screening in Stickler syndrome have been reported. The strongest evidence stems from the UK “Cambridge protocol,” advocating 360° prophylactic cryotherapy to the ora serrata in genetically confirmed type-1 SS, which markedly reduces retinal detachment risk. Laser-based alternatives, such as encircling or extended vitreous base laser, are applied in some centers with favorable but smaller, non-randomized series.

In North America, AAO and AAPOS guidelines recommend lifelong dilated examinations for all SS patients, with prophylactic treatment reserved for those with a confirmed COL2A1 mutation or family/personal RD history, ideally in specialized pediatric retina centers. Many European centers continue to rely on surveillance-only approaches with 6–12-monthly examinations and strong emphasis on patient and family education. Thus, no universally accepted international screening protocol exists, but most strategies converge on early identification of high-risk genotypes, frequent pediatric screening, and prompt treatment or referral when indicated.

Our study does not provide definitive guidance on the optimal screening interval for RD in SS patients. The challenge lies in balancing the prevention of unnecessary ophthalmologist visits while ensuring timely detection of RE. For adults and older children, patient education about RD symptoms can be an effective strategy, although the definition of “older children” varies individually and should be tailored to each patient’s maturity level. Age-appropriate information about symptoms should be provided to enhance patient awareness. Younger children, who cannot be expected to reliably report symptoms, require periodic screening.

An important factor in determining the appropriate screening interval is the high prevalence of myopia, often of significant degree, in SS patients. This refractive error is typically congenital, severe, and frequently accompanied by a substantial astigmatic component. These characteristics underscore the need for a carefully considered approach to screening that accounts for both age and individual risk factors, particularly the degree of myopia present.

The high prevalence of myopia in SS patients necessitates early and regular refraction measurements, starting from 1 year of age. These periodic visits for myopia assessment provide an opportunity to simultaneously examine the retina. Based on our findings and considering the limitations of our study, we suggest a flexible screening protocol that takes into account individual risk factors. For high-risk patients (those with a positive family history of SS, COL2A1 pathogenic variant, and higher myopic refractive error), more frequent follow-ups may be beneficial. However, the optimal interval between screenings requires further investigation through prospective studies. We do not advocate for screening before 1 year of age, as the youngest patient in our cohort experienced RD at 5 years old, and the reliability of ophthalmological examinations in very young children is limited (Table 4).

**Table 4:** Consideration point for screening Sticklers syndrome patients in clinical practice.

Recommendation	Details
Lifetime advice	Visit an ophthalmologist when having symptoms of a retinal detachment
1–10 years old	Periodical follow-up every 6 months
10+ years old	Periodical follow-up every year
Stop follow-up	When patient is able to recognize symptoms of a retinal detachment
Consider earlier switch to yearly follow-up if:	–No (high) myopia
	–No positive family history for Sticklers syndrome
	–No identified COL2A1 pathogenic mutation

**Table 5:** Subgroup details.

Group	Subgroup	Details
35 SS patients with COL2A1 PV	30 patients	Genetically confirmed SS patients with a PV in COL2A1
	5 patients	Clinically diagnosed with SS but not (yet) genetically confirmed, however with affected family member(s) with genetically confirmed COL2A1 PV
43 other identified SS patients	18 patients	Clinically diagnosed with SS in whom up-to-date genetic testing could not confirm SS based on a known PV
	3 patients	SS patients with genetically confirmed PV in COL9A1, COL9A2, COL9A3, COL11A1
	22 patients	Clinical diagnosis of SS without genetic testing and without positive family history of genetically confirmed SS

Our study reveals a significant prevalence of RE among SS patients. Through multivariate survival analysis, we identified several potential risk factors for RE, including a positive family history, COL2A1 PV, and a higher degree of myopic refractive error. To mitigate vision loss in SS patients, we emphasize the critical importance of prompt ophthalmological evaluation upon the onset of RD symptoms. For younger patients or those who may have difficulty recognizing these symptoms reliably, we advocate for periodic ophthalmological screening. Our findings underscore the need for heightened vigilance in young SS patients presenting with a combination of risk factors, particularly a positive family history of SS, COL2A1 PV, and higher myopic refractive error. By implementing a targeted approach to screening and patient education, we aim to optimize early detection and intervention for RE in the SS population, potentially leading to improved long-term visual outcomes. This strategy is crucial for preserving vision and enhancing the quality of life for individuals affected by this connective tissue disorder.

## Summary statement

This is a retrospective cohort study of 78 patients with Stickler syndrome to identify risk factors for the development of retinal detachment. A positive family history of Stickler syndrome, COL2A1 pathogenic variant and higher myopic refractive error are associated with an increased risk of retinal detachment.

## Funding

None.

## Ethical approval

Ethical approval for this study was obtained from the medical ethical research committee of our institution, and informed consent was not required for this retrospective analysis.

## Declaration of competing interest

None declared.
